# Development of Recombinant *Lactococcus lactis* Displaying Albumin-Binding Domain Variants against Shiga Toxin 1 B Subunit

**DOI:** 10.1371/journal.pone.0162625

**Published:** 2016-09-08

**Authors:** Petra Zadravec, Lucie Marečková, Hana Petroková, Vesna Hodnik, Milica Perišić Nanut, Gregor Anderluh, Borut Štrukelj, Petr Malý, Aleš Berlec

**Affiliations:** 1 Department of Biotechnology, Jožef Stefan Institute, Jamova 39, SI-1000, Ljubljana, Slovenia; 2 The Chair of Pharmaceutical Biology, Faculty of Pharmacy, University of Ljubljana, Aškerčeva 7, SI-1000, Ljubljana, Slovenia; 3 Laboratory of Ligand Engineering, Institute of Biotechnology CAS, v. v. i., BIOCEV Research Center, Průmyslová 595, 252 50, Vestec, Czech Republic; 4 Department of Biology, Biotechnical Faculty, University of Ljubljana, Jamnikarjeva 101, 1000, Ljubljana, Slovenia; 5 National Institute of Chemistry, Hajdrihova 19, 1000, Ljubljana, Slovenia; University of Groningen, NETHERLANDS

## Abstract

Infections with shiga toxin-producing bacteria, like enterohemorrhagic *Escherichia coli* and *Shigella dysenteriae*, represent a serious medical problem. No specific and effective treatment is available for patients with these infections, creating a need for the development of new therapies. Recombinant lactic acid bacterium *Lactococcus lactis* was engineered to bind Shiga toxin by displaying novel designed albumin binding domains (ABD) against Shiga toxin 1 B subunit (Stx1B) on their surface. Functional recombinant Stx1B was produced in *Escherichia coli* and used as a target for selection of 17 different ABD variants (named S1B) from the ABD scaffold-derived high-complex combinatorial library in combination with a five-round ribosome display. Two most promising S1Bs (S1B22 and S1B26) were characterized into more details by ELISA, surface plasmon resonance and microscale thermophoresis. Addition of S1Bs changed the subcellular distribution of Stx1B, completely eliminating it from Golgi apparatus most likely by interfering with its retrograde transport. All ABD variants were successfully displayed on the surface of *L*. *lactis* by fusing to the Usp45 secretion signal and to the peptidoglycan-binding C terminus of AcmA. Binding of Stx1B by engineered lactococcal cells was confirmed using flow cytometry and whole cell ELISA. Lactic acid bacteria prepared in this study are potentially useful for the removal of Shiga toxin from human intestine.

## Introduction

Infections with Shiga toxin (Stx)-producing bacteria, such as Stx-producing *Escherichia coli* (STEC) and *Shigella dysenteriae*, cause diarrhea and hemorrhagic colitis in human, and may further develop into a life-threatening hemolytic uremic syndrome (HUS), characterized by microangiopathic hemolytic anemia, thrombocytopenia, and acute renal failure [1, 2]. Shiga toxin, the major virulence factor of STEC, can be produced as Stx1 variants (Stx1 and Stx1c), Stx2 variants (Stx2, Stx2c, Stx2d, Stx2e, Stx2f) or both variants in different combinations [3]. Stx produced by *S*. *dysenteriae* is almost identical to Stx1 produced by *E*. *coli*. All members of Stx family are composed of monomeric enzymatically active A subunit (StxA), which is non-covalently attached to pentameric B subunit (StxB). The latter is responsible for binding to globotriaosylceramide receptor (Gb_3_) on the cell surface, or to globotetraosylceramide (Gb_4_) receptor in the case of Stx2e [3].

Human infection with STEC is mainly associated with ingestion of contaminated food, and can quickly spread among humans causing massive outbreaks of STEC diseases. After ingestion, STEC pass through gastrointestinal (GI) tract, where they colonize the lower GI tract and release Stx into the gut lumen [1]. Stx is absorbed by the intestinal epithelium into the blood circulation and targets tissues expressing Gb_3_ receptor [4]. Once bound, the toxin is very effectively internalized into the cells by endocytosis and transported through the retrograde pathway, whereby active A subunit is cleaved by furin and translocated to cytosol, where it functions as a highly specific N-glycosidase, inhibiting protein synthesis and causing cell death [5, 6].

Currently, there is no specific treatment for patients with developed HUS. Recommended management relies on supportive therapy that includes fluid and electrolyte balance, nutritional support, management of hypertension and renal transplantation [4]. Because the conventional antibiotic treatment of STEC infections increases the risk of developing HUS by induction of Stx expression and toxin release into the gut, there is a need for the development of new therapies. Various potential therapeutic strategies are currently under development, and include compounds directed against STEC, Stx receptor analogues, receptor synthesis inhibitors, antitoxin antibodies, inhibitors of toxin transport, processing and function, natural products and also novel antimicrobial therapies (reviewed in [7, 8]). Monoclonal antibodies against Stx1 and Stx2 are currently furthest in development [7].

As an alternative to conventional monoclonal antibodies, more than 20 different types of small single-domain non-immunoglobulin (non-Ig) scaffolds are being used to produce binding proteins against more than a hundred different targets [9]. Compared to antibodies, engineered non-Ig scaffolds are stable, robust and soluble monomeric proteins, that lack disulfide bonds, are easily and inexpensively produced in large amounts in bacteria, exhibit effective tissue penetration, rapid distribution and elimination, can be easily modified by conjugation or gene fusion technology and can exert multivalency or multispecificity [9, 10]. Among them, a highly complex combinatorial library, derived from the three-helix bundle of the albumin-binding domain (ABD) scaffold of streptococcal protein G has been used successfully for the selection of human interferon gamma binders [11], interleukin (IL)-23 receptor antagonist [12] and binders of human prostate secretory protein 94 (PSP94) [13]. In this work we applied the ABD scaffold library for the selection of unique binders of Stx1B.

Probiotics, including *E*. *coli* Nissle 1917 [14], *E*. *coli* 1307 [15], and several *Lactobacillus* strains [16] were reported as effective inhibitors of growth of STEC. Lactic acid bacteria (LAB) are often used as probiotics and are, because of their safety, also considered for genetic engineering and delivery of therapeutic proteins to the human intestine. We have previously demonstrated effective display of two non-Ig scaffolds, Affibodies [17] and DARPins [18], on the surface of recombinant or nonrecombinant lactic acid bacteria (LAB), by using the C terminal part of the lactococcal AcmA protein (cA) containing the lysine motif (LysM) domain as the cell wall anchor [19–22]. Engineered probiotic LAB with surface displayed Stx-binding protein could be a promising candidate for treating infections caused by STEC or *S*. *dysenteriae*. A similar approach has already been used in the development of recombinant *E*. *coli* bacteria with an engineered oligosaccharide biosynthesis pathway that resulted in the production of Stx receptor mimic on the bacterial surface [23, 24].

The goal of the present study was to engineer recombinant LAB *Lactococcus lactis* capable of binding Stx1B, by displaying binding proteins against Stx1B on the surface of *L*. *lactis*. We chose StxB as a target for selecting binding proteins against Stx, because it is capable of binding to the receptor and is not toxic by itself. We have expressed, purified and characterized recombinant Stx1B, and used it for the development of ABD-derived binding proteins S1Bs against Stx1B. S1Bs were biochemically, biophysically and functionally characterized, displayed on the surface of *L*. *lactis* and their ability to bind Stx1B was confirmed.

## Materials and Methods

### Bacterial strains, media and culture conditions

The bacterial strains used in this study are listed in Table 1. *E*. *coli* strains DH5α, BL21 (DE3) and BL21 (DE3) BirA were grown at 37°C, unless otherwise stated, with aeration in lysogeny broth (LB) medium supplemented with 50 μg/mL kanamycin. *L*. *lactis* NZ9000 was grown in M-17 medium (Merck) supplemented with 0.5% glucose (GM-17) and 10 μg/mL of chloramphenicol at 30°C without aeration.

**Table 1:** Strains, plasmids, gene and primers used in this study.

Strain, plasmid, gene or primer	Relevant features or sequence (5’– 3’)	Reference
Strains		
*E*. *coli*		
DH5α	endA1 glnV44 thi-1 recA1 relA1 gyrA96 deoR F- Φ80dlacZΔM15 Δ(lacZYA-argF)U169, hsdR17(rK- mK+), λ–	Invitrogen
BL21 (DE3)	F–ompT gal dcm lon hsdSB (rB- mB-) λ(DE3)	Novagen
BL21 (DE3) BirA	BL21 (DE3) with biotin ligase gene	Novagen
*L*. *lactis* NZ9000	MG1363 nisRK ΔpepN	[28–31]
Plasmids		
pET28b(+)	Kanr, *E*. *coli* expression vector	Novagen
pNZ8148	pSH71 derivative, PnisA, Cmr, nisin-controlled expression	[28–31]
pSDLBA3b	pNZ8148 containing gene fusion of spUsp-LEIS, b-dom and cA	[17]
pET28-Stx1B	pET28b containing Stx1B gene	This work
pET28- H6-TolA-Avi	pET28b containing tolA gene with AviTag on C-terminus	[12]
pET28-H6-S1Bx-TolA-Avi	pET28b containing gene fusion of different variants of S1B clones with TolA and AviTag	This work
pET28-H6-ABDwt-TolA-Avi	pET28b containing gene fusion of ABDwt with TolA and AviTag	
pSD-S1B22	pNZ8148 containing gene fusion of Usp45 signal peptide, S1B22 and cA	This work
pSD-S1B26	pNZ8148 containing gene fusion of Usp45 signal peptide, S1B26 and cA	This work
pSD-ABDwt	pNZ8148 containing gene fusion of Usp45 signal peptide, ABDwt and cA	This work
pSD-H6-ABDwt	pNZ8148 containing gene fusion of Usp45 signal peptide, H6 tag, ABDwt and cA	This work
Gene		
Stx1B	CCATGGCAAAAAAAACATTATTAATAGCTGCATCGCTTTCATTTTTTTCAGCAAGTGCGCTGGCGACGCCTGATTGTGTAACTGGAAAGGTGGAGTATACAAAATATAATGATGACGATACCTTTACAGTTAAAGTGGGTGATAAAGAATTATTTACCAACAGATGGAATCTTCAGTCTCTTCTTCTCAGTGCGCAAATTACGGGGATGACTGTAACCATTAAAACTAATGCCTGTCATAATGGAGGGGGATTCAGCGAAGTTATTTTTCGTCTCGAG	This work
Primer		
setB-rev	ACCGCGGATCCAGGTAA	[11, 12]
EWT5-ABDforN1	TTCCTCCATGGGTATGAGAGGATCGCATCACCATCACCATCACCTGGCGGAAGCTAAAGTCTTAGCTAAC	[13]
EWT5-ABDforN2	TTCCTCCATGGGCAGCAGCCATCACCATCACCATCACCTGGCGGAAGCTAAAGTCTTAGCTAAC	[13]
ABD-F	GGATCCCTGGCGGAAGCTAAAGTC	This work
S1B22-R	GAATTCAGGTAAACGAGCTAAAATAGCATCTATC	This work
S1B26-R	GAATTCAGGTAACGCAGCTAAAATCCAATC	This work
ABDwt-R	GAATTCAGGTAATGCAGCTAAAATTTCATCTATC	This work
ABDH6-F	AGGATCCCATCACCATCACCATCAC	This work

### Preparation of recombinant Stx1B subunit

A gene for Stx1B was designed (Table 1), synthesized *de novo* by ATG Biosynthetics (Merzhausen, Germany) and cloned to plasmid pET28b using NcoI/XhoI restriction sites, yielding pET28-Stx1B. Overnight culture of *E*. *coli* BL21 (DE3) harboring plasmid pET28-Stx1B was diluted (1:100) in 1 L of fresh LB medium and grown to optical density A_600_ = 3.5–4.0. Expression of fusion protein Stx1B with hexa-histidine (H6) tag was induced by addition of 1 mM isopropyl β-D-1-thiogalactopyranoside (IPTG) for 3 h at 28°C. The culture was centrifuged at 5000 × g for 15 min and the pellet resuspended in 30 mL of equilibration/wash (Eq/W) buffer (50 mM NaH_2_PO_4_, 300 mM NaCl, pH 7.0). The cells were lysed with a cycle of freezing and thawing, and with 3 fold 5 min sonication with a UPS200S sonifier (Hielscher, Teltow, Germany). After cell lysis, the suspension was centrifuged at 15000 × g for 20 min and the supernatant stored. Inclusion bodies were dissolved in Eq/W buffers with increasing concentrations of guanidinium HCl (1M, 3M and 6M) for 6 h or overnight at 4°C, followed at each step by centrifugation and supernatant removal. Stx1B-H6 soluble in Eq/W with 6 M guanidinium HCl was isolated with BD Talon metal affinity resin (BD Biosciences) according to the manufacturer’s instructions, using batch/gravity-flow column purification and imidazole elution (elution buffer: 45 mM NaH_2_PO_4_, 270 mM NaCl, 5.4 M guanidinium HCl, 150 mM imidazole, pH 7.0). Fractions containing pure Stx1B were pooled and stored. We screened different refolding conditions according to [25–27]. Recombinant Stx1B was refolded by 100-fold rapid dilution in solubilization buffer (50 mM Tris-HCl with 0.5 M arginine and 0.01% Brij-35, pH 7.5).

### Determination of molecular weight of Stx1B subunit

The molecular weight and oligomerization status of Stx1B were determined using analytical gel filtration chromatography (1.2 × 60 cm, 7 mL/h flow rate with 15 min fraction collection time) on a polyacrylamide gel Bio-Gel P-100 (Bio Rad, Hercules, USA), due to Stx1B cross-reactivity with a Superdex column (GE Healthcare), for size exclusion chromatography. Six proteins of 14.4–97 kDa (Amersham Low molecular weight Calibration Kit, GE Healthcare) were used as standards.

### *In vitro* binding of Stx1B to globotriaosylceramide (Gb_3_) receptor

Binding of Stx1B to its natural receptor Gb_3_ was determined by enzyme-linked immunosorbent assay (Gb_3_ ELISA) as described [32]. Receptor Gb_3_ was purchased from Matreya LLC (PA, USA) and dissolved in chloroform/methanol (2:1). 100 μL of Gb_3_ solution with concentration 10 μg/mL was used to coat Nunc PolySorp Strips (Thermo Fisher Scientific) overnight in laminar flow to evaporate chloroform/methanol. After washing with phosphate buffer saline with 0.05% Tween 20 (PBST) and blocking with 2% bovine serum albumin (BSA) in PBST, 100 μL of serial twofold dilutions of recombinant Stx1B in triplicate (starting with 1 μg/mL in 0.2% BSA in PBST) were added to the wells and incubated for 1 h. Bound Stx1B-H6 was detected with primary THE^™^ His tag Antibody (GenScript, NJ, USA) (dilution 1:2000 in 0.2% BSA in PBST) and with horseradish peroxidase (HRP)-conjugated goat anti-mouse IgG secondary antibody (Merck Millipore, Darmstadt, Germany) (dilution 1:5000 in 0.2% BSA in PBST). The color was developed by the addition of 100 μL substrate buffer (150 mM Na_2_HPO_4_, 50 mM citric acid, pH 6.0) and 100 μL 3,3',5,5'-tetramethylbenzidine (TMB) substrate (Sigma-Aldrich, MO, USA) for 15 min at room temperature. The reaction was terminated by the addition of 50 μL 2 M H_2_SO_4_ and absorbances were read at 450 nm using Infinite M1000 (Tecan, Salzburg, Austria). No Stx1B was added to 0.2% BSA in PBST in control wells (zero concentration), while all the other steps were performed as described above. All samples were measured in triplicates.

### Internalization of recombinant Stx1B by HeLa cells

HeLa cells (American Type Culture Collection) were grown in Dulbecco's Modified Eagle Medium (DMEM) supplemented with 10% fetal bovine serum (FBS), 1% GlutaMAX and pen-strep in 24-wells plates on coverslips. Fluorescein isothiocyanate (FITC)-labelled Stx1B (10 μg/mL in 300 μL of fresh medium) was added to the cells and incubated for 1 h on 37°C. The cells were then washed with PBS, fixed with 4% paraformaldehyde (PFA) in PBS for 15 min and permeabilized with 0.1% triton X-100 in PBS for 10 min. Non-specific staining was blocked with 3% BSA in PBS for 1 h. Golgi apparatus (GA) was labelled with mouse monoclonal anti-human Golgin-97 primary antibody (0.4 μg/ml in 3% BSA for 1 h, Life Technologies, CA, USA) and with Alexa Fluor 555-conjugated donkey anti-mouse secondary antibody (1:1000 in 3% BSA for 1 h, A-31570, Life technologies). Coverslips were mounted with ProlongGold Antifade reagent with 4',6-diamidino-2-phenylindole (DAPI; Invitrogen). Immunostained cells were visualized with LSM-710 confocal microscope (Carl Zeiss, Germany), and images were acquired and processed using ZEN 2010 B SP1 software (Carl Zeiss).

### Selection of Stx1B binders by ribosome display

The combinatorial DNA library of ABD protein was generated as described [11, 12], *in vitro* transcribed and the resulting mRNA was translated using *E*. *coli* extract (EasyXpress Protein Synthesis Mini Kit, QIAGEN, Germany). The translated products were used for the selection of Stx1B binders by ribosome display. Preselection of BSA binders was performed in wells coated with 3% BSA before the first round of the selection. The five rounds of ribosome display selections were performed in Maxisorp (NUNC, Denmark) microtiter plates coated with decreasing concentrations of recombinant Stx1B (round 1 and 2: 25 μg/mL, round 3: 10 μg/mL, round 4: 4 μg/mL, round 5: 1 μg/mL) and blocked with 3% BSA. All incubation and washing steps were performed as described [12] and after each round, mRNA was eluted from the bound ribosome complex with elution buffer (50 mM Tris-acetate, pH 7.5, 150 mM NaCl, 50 mM EDTA) containing 50 μg/mL of *Sacharomyces cerevisiae* RNA. Purified RNA was transcribed into cDNA using reverse transcription with setB-rev primer (Table 1). Double-stranded DNA was produced by PCR using EWT5–ABDforN1 and setB-rev primers (Table 1) and fused with T7 promoter, RBS and truncated *tolA* sequences for the next round of *in vitro* translation. After the last round of the selection the final PCR product was amplified with EWT5–ABDforN2 (Table 1) primer instead of the EWT5–ABDforN1 primer, and cloned to pET28-H6-TolA-Avi plasmid (Table 1) via NcoI/BamHI sites, yielding pET28-H6-S1B-TolA-Avi. The resulting plasmid mixture was transformed into *E*. *coli* DH5α, and plasmid DNA from randomly picked colonies was sequenced.

### Sequence analysis and clustering of selected S1B binders

DNA sequencing of S1B variants was carried out by the Centre for DNA Sequencing of the Institute of Microbiology of the ASCR, v.v.i. (Prague, Czech Republic) or GATC Biotech (Constance, Germany). Amino acid sequences of selected clones were aligned and a similarity tree was constructed using Molecular Evolutionary Genetics Analysis tool (MEGA), version 6.0.6 (http://www.megasoftware.net/). Randomized sequences between residues 20 and 46 were compared, as the N-terminal amino acid positions 1–19 were non-randomized.

### ELISA screening of Stx1B binders

S1B clones with unique (previously unreported) sequences were transformed into *E*. *coli* BL21 (DE3) BirA (Table 1) and expressed for 4 h in 2.5 mL cultures with the addition of 1 mM IPTG and 50 μM D-biotin. Bacteria were pelleted at 15000 × g for 5 min and resuspended in 0.01% PBST with 200 μg/mL lysozyme. Cells were lysed with three cycles of freezing at -80°C and thawing at 37°C for 30 min. Cell lysate was centrifuged for 15 min and 50 μL of supernatant was loaded to a PolySorp microtiter plate (Nunc), previously coated with 10 μg/mL of recombinant Stx1B and blocked with 1% BSA (Carl Roth GmbH, Karlsruhe, Germany). After 1 h incubation at RT, the plate was washed five times with PBST, and ABD-binders were detected with HRP-conjugated streptavidin (1:5000 in 1% BSA/PBST). The color was developed with the addition of 0.5 mg/mL o-phenylenediamine (OPD, Sigma-Aldrich, St. Louis, USA) and 0.01% H_2_O_2_ in 0.1 M citrate buffer (pH 5.0) for 5 min. The reaction was stopped with the addition of 2 M H_2_SO_4_ and absorbance read at 492 nm. Lysate containing the wild-type albumin binding domain (ABDwt) in fusion with TolA protein was loaded to wells coated with 10 μg/mL of human serum albumin (Abcam, Cambridge, UK) to serve as a positive control. Negative background was recorded in wells loaded with 1% BSA/ PBST without S1B-TolA lysate. All samples were measured in triplicates.

### Expression and purification of selected H6-S1B-TolA-Avi variants

Selected H6-S1B-TolA-Avi variants were expressed in 200 mL cultures as described above. Cells were harvested by centrifugation at 5000 × g for 20 min, resuspended in 10 mL binding buffer (20 mM Na-phosphate, 500 mM NaCl, 10 mM imidazole, pH 7.4) and lysed by sonication for 3 min. Lysates were centrifuged at 10000 × g for 20 min and supernatants were kept. Fusion proteins were purified by fast protein liquid chromatography (Äkta Purifier, GE Healthcare) on 1 mL HisTrap HP columns (GE Healthcare) or BD Talon metal affinity resin (BD Biosciences, Palo Alto, USA) using batch/gravity-flow column purification and imidazole elution according to the manufacturer instructions. Eluted fractions were analyzed by SDS-PAGE, pooled and concentrated by ultrafiltration using Amicon Ultra (Merck Millipore; Darmstadt, Germany). Purified H6-S1B-TolA-Avi fusion proteins were dialyzed against PBS and used for ELISA tests, surface plasmon resonance analysis and microscale thermophoresis.

### Assessment of binding of S1B variants to Stx1B by ELISA

The microtiter plate was coated overnight at 4°C with 10 μg/mL of recombinant Stx1B in coating buffer (100 mM Na-bicarbonate/carbonate, pH 9.6). Wells were washed with PBST and blocked with 1% BSA in PBST for 2 h at RT. Serially diluted H6-S1B-TolA-Avi fusion proteins or ABDwt control in 1% BSA/PBST were loaded for 1 h. Following washing, bound S1B clones were detected with the addition of HRP-conjugated streptavidin (dilution 1:5000 in 1% BSA/PBST). The color was developed with 0.5 mg/mL OPD and 0.01% H_2_O_2_ in 0.1 M citrate buffer (pH 5.0) for 5 min. The reaction was stopped by the addition of 2 M H_2_SO_4_ and absorbance read at 492 nm. All samples were measured in triplicates.

### Assessment of binding of S1B variants to Stx1B by surface plasmon resonance (SPR)

SPR measurements were performed with Biacore T100 (GE Healthcare) at 25°C. In the first setup we tested the binding of S1B variants prepared in fusion with TolA spacer protein and AviTag to Stx1B captured on a Series S sensor chip CM5 (Biacore, GE Healthcare). CM5 sensor chip was activated using Amine coupling Kit (GE Healthcare) according to the manufacturer’s instructions. For immobilization of Stx1B standard PBS buffer (137 mM NaCl, 2.7 mM KCl, 10 mM Na_2_HPO_4_, 1.8 mM KH_2_PO_4_, pH 7.4) was used as running buffer. Stx1B was diluted into 10 mM sodium acetate buffer, pH 5.0, to a final concentration of 20 μg/mL, and injected over the second flow cell. The final immobilization level was approximately 890 response units (RU). The first flow cell was empty and served as a reference cell to control the level of non-specific binding. All experiments were performed with running buffer PBS with 300 mM NaCl (300 mM NaCl, 2.7 mM KCl, 10 mM Na_2_HPO_4_, 1.8 mM KH_2_PO_4_, pH 7.4, 0.005% P20). Each compound was injected for 2 min at a flow-rate of 20 μL/min, and dissociation was monitored for 3 min. Regeneration of the sensor surface was achieved with two 30 s pulses of 50 mM NaOH. The following concentrations were used for titration: 0, 15.625, 31.25, 62.5, 125, 250, 500, 1000, 2000 nM; concentration 62.5 nM was repeated at the end of the concentration series.

In the reverse setup H6-S1B22-TolA-Avi or H6-S1B26-TolA-Avi variants were immobilized on a sensor chip CM5. CM5 sensor chip activation and immobilization of H6-S1B-TolA-Avi variants was performed as described above, except that two injections were needed to reach the final immobilization level of approximately 1100 RU. All experiments were performed with running buffer PBS with 300 mM NaCl (300 mM NaCl, 2.7 mM KCl, 10 mM Na_2_HPO_4_, 1.8 mM KH_2_PO_4_, pH 7.4, 0.005% P20). Each compound was injected for 1 min at a flow-rate of 10 μL/min, and dissociation was monitored for 60 s. Regeneration of the sensor surface was achieved with 25 mM NaOH for 6 s at a flow-rate of 20 μL/min. The following concentrations were used for titration: 62.5, 125, 250, 500, 1000, 2000, 4000 nM and the concentration of 500 nM was repeated at the end of the concentration series. Each titration was performed in triplicate. The obtained data was evaluated using Biacore T100 Evaluation software. The sensorgrams were reference and blank subtracted and the Steady State Affinity model was applied to calculate the affinity constant (*K*_d_). The average of three repeated experiments was used for final *K*_d_ determination.

### Assessment of binding of S1B variants to Stx1B by microscale thermophoresis (MST)

Purified Stx1B in PBS buffer (pH 7.4) was fluorescently labelled according to the manufacturer’s instructions (L001^™^ Monolith NT.115 Protein Labelling Kit RED-NHS, NanoTemper Technologies GmbH, Germany). The stock solution of labelled Stx1B was diluted in PBS buffer with 300 mM NaCl and 0.1% Tween 20 to a final concentration of 10 nM. Sixteen serial dilutions of purified non-labelled S1B22 and S1B26 were prepared in standard PBS buffer, starting with concentrations of 35 μM and 45 μM, respectively. Serial dilutions of S1B variants were mixed 1:1 with a solution of 10 nM labelled Stx1B to a final volume of 20 μL per dilution. Prepared mixtures were incubated for 5 min at room temperature and centrifuged for 5 min at 15,000 g to remove any aggregates. The samples were filled into the Premium coated capillaries (NanoTemper Technologies GmbH, Germany). Measurements were carried out with the Monolith NT.115 instrument (NanoTemper Technologies GmbH) at 25°C using 90% LED power and 20% MST power. Data analysis was completed with NanoTemper analysis software using fluorescence change for *K*_d_ determination. The SDS-denaturation test was performed to confirm that the fluorescence change is the consequence of specific binding of ligand to the target and not due to loss of fluorescence caused by ligand-induced surface absorption or aggregation. The average of three repeated experiments was considered for final *K*_d_ calculation.

### Fluorescence-based thermal shift assay (TSA)

The thermal stability of S1B proteins was tested in PBS and 50 mM Tris, 300 mM NaCl, pH 8.0 (T50N300) buffers with the addition of 5× Sypro Orange dye (Sigma-Aldrich, St. Luis, USA) to the 25 μL total volume. TSA was performed with the real-time PCR Detection System CFX touch (BIO-Rad Laboratories, Hercules, USA) according to [12]. The data were analyzed with CFX Manager Software and the melting temperatures (Tm) determined using the first derivative spectra.

### Influence of S1B binders on internalization of Stx1B into HeLa cells

S1B22, S1B26 and ABDwt (final concentration 100 μg/ml) were preincubated with Alexa Fluor 488-labelled recombinant Stx1B (Alexa Fluor 488^®^ Protein Labeling Kit, Thermo Fisher Scientific, Massachusetts, USA—final concentration 5 μg/ml) for 1 h in complete DMEM medium at 37°C. Stx1B, ABDwt, S1B22 or S1B26 were used alone as corresponding controls. Afterwards, the mixtures of protein and Alexa Fluor 488-Stx1B were added to HeLa cells grown in 24 well plates (flow cytometry) or on coverslips (fluorescence microscopy) and incubated for 1 h at 37°C. After incubation, the media were discarded and cells were washed with PBS and prepared for further analysis.

For flow cytometric analysis cells were detached by mild trypsinization (TripleSelect, Life Technologies) washed three times with PBS and measurements were performed on a BD FACS Calibur instrument (Beckton Dickinson, Franklin Lakes, NJ) as described in [18] using BD CellQuest Pro software for data acquisition and analysis (Beckton Dickinson).

For fluorescence microscopy the coverslips-grown cells were either labeled with PKH26 dye for general cell membrane labeling or stained with antibodies for GA labelling. Membrane labelling was performed with Red Fluorescent Cell Linker Kit containing PKH26 dye (Sigma-Aldrich, St. Louis, USA) following manufacturer’s instructions. Afterwards, the cells were fixed with 2% PFA and washed with PBS. For labeling with antibodies cells were first fixed in 2% PFA, permeabilized with 0.1% Triton X-100 and incubated for 1 h in 3% BSA in PBS for blocking of non-specific staining. For labeling of GA primary mouse monoclonal anti-Golgin-97 (CDFX) antibody (1:100 in 3% BSA in PBS, Santa Cruz Biotechnology, Dallas, USA, sc-59820) and polyclonal Goat anti-Mouse Secondary Antibody conjugated with Alexa Fluor 633 (1:1000 in 1% BSA in PBS, Thermo Fisher Scientific, A-21052) were used. Cells were washed three times with PBS after every of the above mentioned steps. After the last washing step cells were stained with DAPI (Sigma-Aldrich, St. Louis, USA) and mounted with Prolong Gold Antifade Reagent (Life technologies). Immunostained cells were visualized with LSM-710 confocal microscope equipped with ZEN 2010 B SP1 software (Carl Zeiss).

### Molecular cloning and expression of S1B-cA variants in *L*. *lactis*

Genes of S1B variants without TolA spacer were amplified with Phusion polymerase (New England Biolabs, Beverly, MA) using pET28-H6-S1Bx-TolA-Avi template and ABD-F primer and the corresponding S1B-R primer (Table 1). The gene for ABDwt was amplified using pET28-H6-ABDwt-TolA-Avi template and ABD-F/ABDwt-R (Table 1) primer pair, while H6 tag was added with ABDH6-F/ABDwt-R primer. All PCR fragments were ligated to pJET 1.2 cloning vector and cloned into plasmid pSDLBA3b, using BamHI and EcoRI restriction enzymes (New England Biolabs). Plasmid DNA was isolated with NucleoSpin Plasmid kit (Macherey and Nagel, Düren, Germany), with an additional lysozyme treatment for *L*. *lactis*. Lactococci were transformed by electroporation using a Gene Pulser II apparatus (Bio-Rad, Hercules, CA) according to MoBiTec GmbH instructions (Goettingen, Germany). Plasmids constructed in the study are listed in Table 1. S1B-cA variants were expressed in *L*. *lactis* NZ9000 in 10 mL cultures. Bacterial suspensions were grown to an A_600_ of 0.8, followed by induction with 25 ng/mL nisin (Fluka) for 3 h [17, 18, 33, 34]. Resulting suspensions were stored at 4°C for flow cytometric analysis or whole cell ELISA test.

### Flow cytometry and whole cell ELISA

For flow cytometric analyses, 10 μL of *L*. *lactis* suspensions were added to Tris-buffered saline (TBS) and centrifuged. Cells were resuspended in TBS with Alexa 488-labelled recombinant Stx1B (20 μg/mL) and incubated at RT with constant shaking at 100 rpm. All washings, centrifugation steps and measurements were performed as described [18, 35].

The whole cell ELISA was carried out according to [36] with a few modifications. *L*. *lactis* suspension with surface-displayed S1B variant or ABDwt was centrifuged and resuspended in PBS to an A_600_ of 1.0. 750 μL of the suspensions were washed twice in PBS and incubated with 500 μL of recombinant Stx1B (20 μg/mL in PBS) for 2 h, followed by additional washing with PBS. Bound toxin subunit was detected with the addition of 200 μL of Anti-Verotoxin I/SLT 1b (antiStx1B) primary antibody (diluted 1:20 in PBS, Abcam, Cambridge, UK) for 1 h, followed by washing and incubation with 200 μL of (HRP)-conjugated Goat anti-mouse IgG secondary antibody (diluted 1:500 in PBS, Merck Millipore, Darmstadt, Germany). All incubations were performed at RT in a tube rotator. After incubation with secondary antibody, cells were washed, first with PBS and then with substrate buffer (150 mM Na_2_HPO_4_, 50 mM citric acid, pH 6.0). Cells were finally resuspended in 1 mL of substrate buffer, and 100 μL of suspensions, or 1:5 dilutions in substrate buffer, were loaded on a microtiter plate. The color was developed with the addition of 100 μL TMB substrate and the reaction stopped after 15 min by the addition of 50 μL of 2 M sulphuric acid. Absorbances were read at 450 nm.

### Statistical analyses

Statistical analyses were performed with GraphPad Prism 5.0 software. Student’s t test was used to compare the significance of differences between samples and control.

## Results and Discussion

### Production of recombinant Stx1B

Recombinant Shiga toxin 1B subunit (Stx1B) fusion protein with hexa histidine (H6) tag was produced in *E*. *coli* as a target for the selection of high affinity binders derived from ABD scaffold by using a novel procedure for its recombinant expression. The gene for Stx1B was synthesized and cloned to pET28b vector. Various expression conditions (growth at 37°C, 30°C and 25°C, induction at optical densities (A_600_) 0.5, 1, 2 and 3.5–4.0) were tested. The highest total amount of Stx1B-H6 expression was achieved by growing the bacteria at 37°C to A_600_ = 3.5–4.0, followed by induction with 1 mM IPTG for 3 h at 28°C. The majority of the fusion protein was produced in the form of inclusion bodies, which were dissolved in 6 M guanidinium HCl and efficiently purified with immobilized metal affinity chromatography (IMAC). We screened different refolding conditions and Stx1B-H6 was effectively solubilized by the rapid dilution method using 0.5 M arginine and 0.01% Brij-35-containing 50 mM Tris buffer, conditions similar to those reported by Oneda and Inouye [27] for another protein. The solubilized product was stored in Tris or PBS buffer for characterization.

### Characterization of recombinant Stx1B

The molecular weight of recombinant Stx1B-H6 was determined by analytical gel filtration chromatography. It was eluted from calibrated column as one sharp symmetrical peak, with a molecular weight corresponding to the pentameric form of recombinant Stx1B.

**Fig 1:**
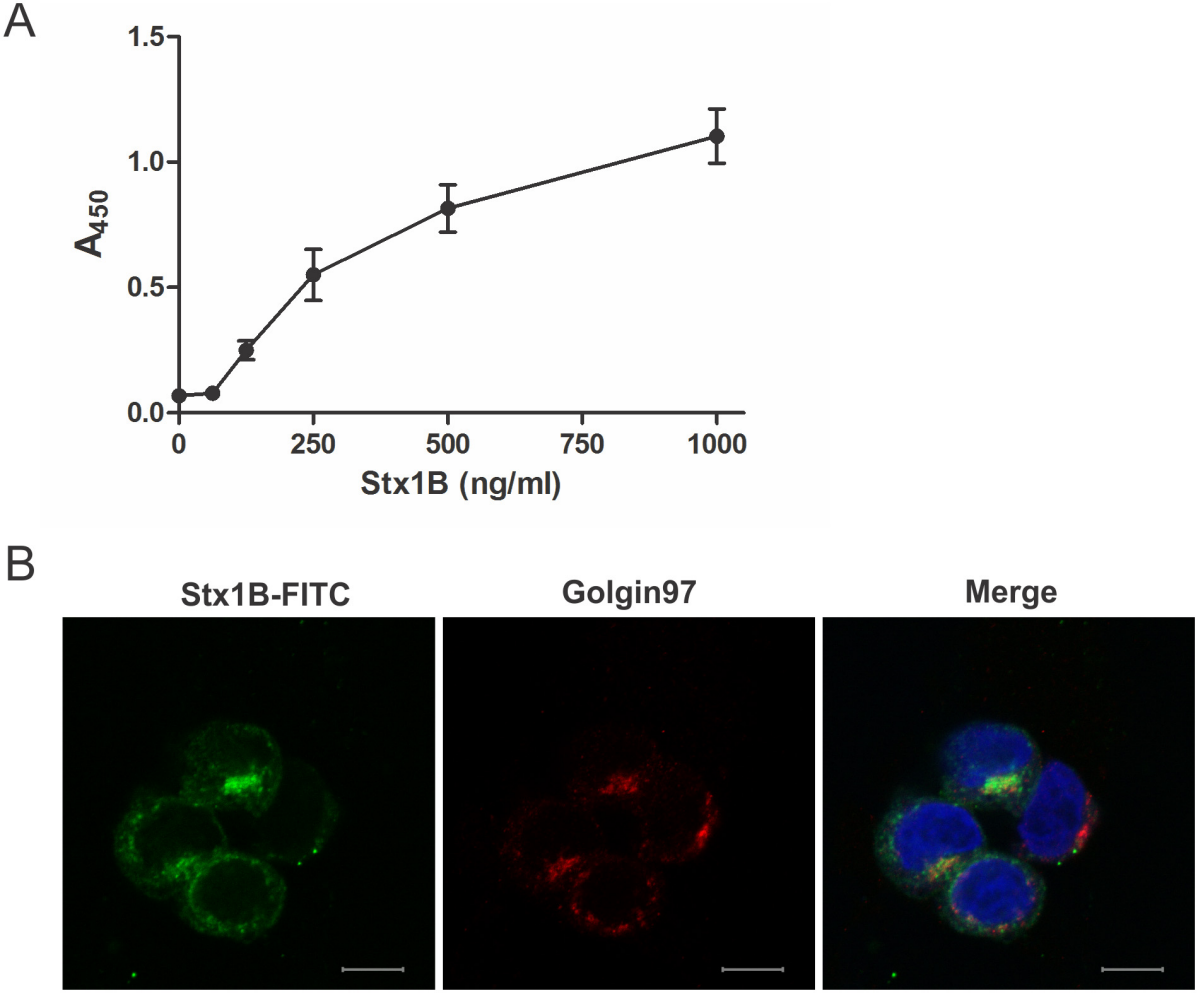
Ability of recombinant Stx1B to bind to Gb_3_ receptor *in vitro*. (A) Binding of serially diluted Stx1B to Gb_3_ as determined by ELISA using THE^™^ His tag antibody. A_450_: Absorbance at 450 nm. Error bars denote standard deviations. (B) Internalization of FITC-labelled Stx1B into HeLa cells. Golgi apparatus was detected with mouse anti-human Golgin-97 primary antibody and Alexa Fluor 555-conjugated donkey anti-mouse secondary antibody (red). DAPI staining was used to label nuclei (blue). Bars = 10 μm.

The functionality of recombinant Stx1B was tested by its ability to bind to immobilized globotriosyl ceramide (Gb_3_) receptor *in vitro*, and by its internalization into HeLa cells, which naturally contain Gb_3_ on the surface. Strong, specific and concentration depended binding of recombinant Stx1B to immobilized Gb_3_ receptor was demonstrated with ELISA (Fig 1A). Stx1B was internalized into the HeLa cells 1 h after co-incubation and was transferred to GA where it co-localized with the GA marker Golgin-97 (Fig 1B), which is in accordance with a previous study [37]. These results showed that recombinant Stx1B was fully functional and suitable as a target for selection of novel high affinity binders.

### Ribosome display selection of Stx1B binders

Smaller antibody variants, i.e. single chain variable fragments (ScFv), against Stx1B were previously selected by screening a phage display library constructed from a naïve human repertoire [38]. However, it was previously observed that, similar to antibodies themselves, ScFvs are not suitable for surface display on lactococcal cells (unpublished data), especially in comparison to non-Ig scaffolds, such as DARPins and Affibodies, possibly due to their larger size and more complex folding. To obtain novel protein binders of Shiga toxin, a highly complex combinatorial library derived from streptococcal albumin-binding domain scaffold was therefore applied. This has been used successfully for selecting high-affinity binders of IFN-γ [11], for development of IL-23 receptor antagonists [12] and for generation of unique binders of human prostate cancer oncomarker [13].

**Fig 2:**
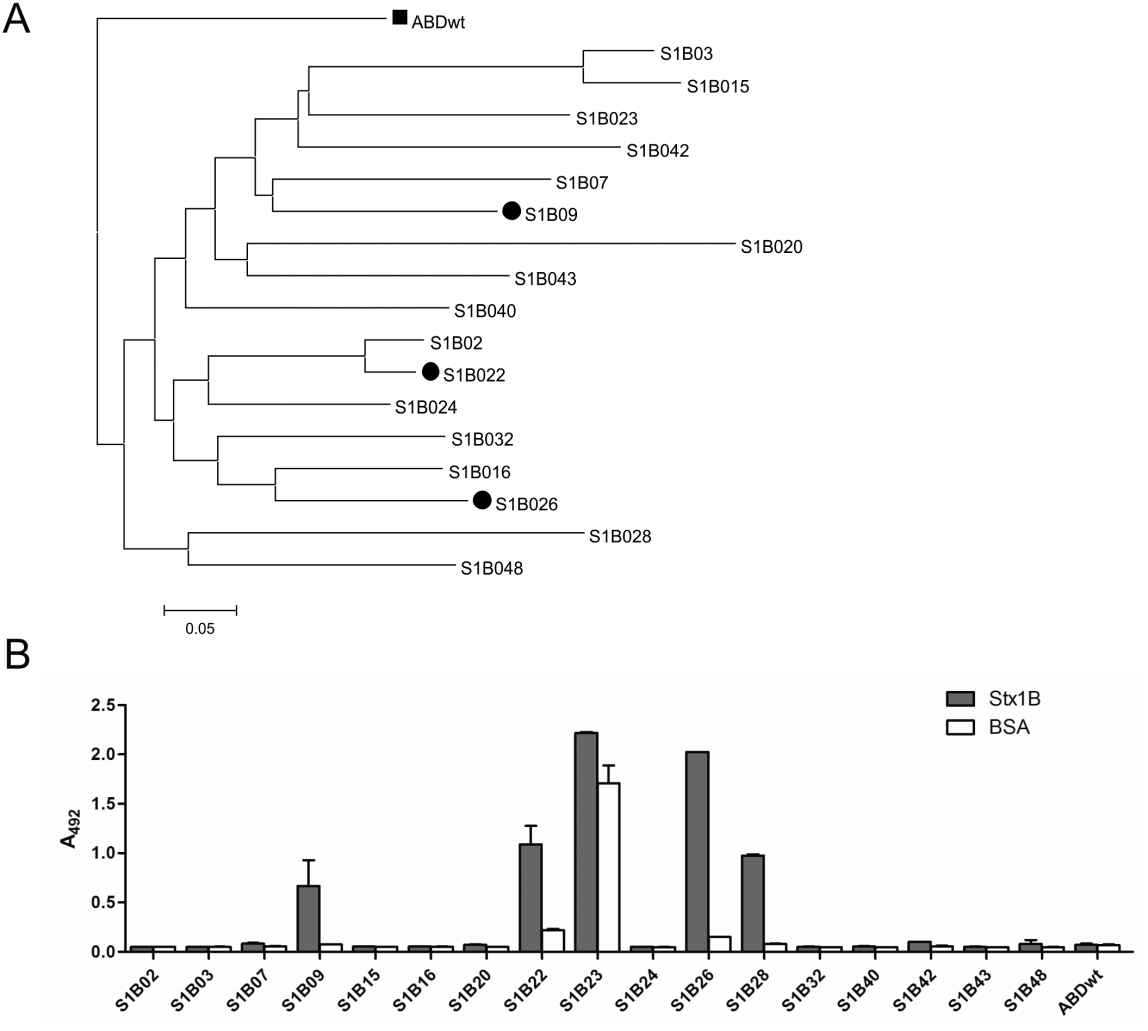
Sequence similarity analysis (A) and binding affinity (B) of 17 S1B binders selected after five rounds of ribosome display. A: The sequence of the parental ABD wild-type domain was used as a root of the tree and is highlighted as a square, while S1B variants selected for more detailed analysis are highlighted as circles. B: S1B binders-containing cell lysates were incubated with immobilized Stx1B (grey bars) or BSA (white bars) and detected with HRP-conjugated streptavidin. Error bars denote standard deviations.

To select unique binders of Stx1B we used the highly-complex combinatorial ABD library [11], [12], [13] and ribosome display. Ribosome display enables the selection of binding proteins by associating ribosome-translated proteins with their parent mRNA in a complex. The mRNA-protein hybrids are selected against an immobilized ligand in multiple selection steps. The mRNA is then reverse transcribed to cDNA and its sequence amplified via PCR [39]. In the present work five cycles of ribosome display selection were performed, yielding a collection of 17 unique Stx1B-binders designated as S1B binders (Fig 2A). The identified S1B binders were produced in *E*. *coli* as fusion proteins with TolA spacer containing AviTag on the C-terminus and a H6 tag on the N-terminus (H6-S1B-TolA-Avi), as reported previously [11–13]. The expression level of the corresponding fusion protein was tested by SDS PAGE and the binding capacity verified by ELISA (Fig 2B). Binders S1B9, S1B22 and S1B26 with TolA-Avi and H6 fusion were selected for further analysis. The S1B28 variant showed a lower level of expression, possibly due to an unintended mutation in a non-randomized part of sequence, while S1B23 bound BSA and was therefore not Stx1B-specific. Both variants were excluded from further characterization.

### Production and characterization of selected S1B binders

**Fig 3:**
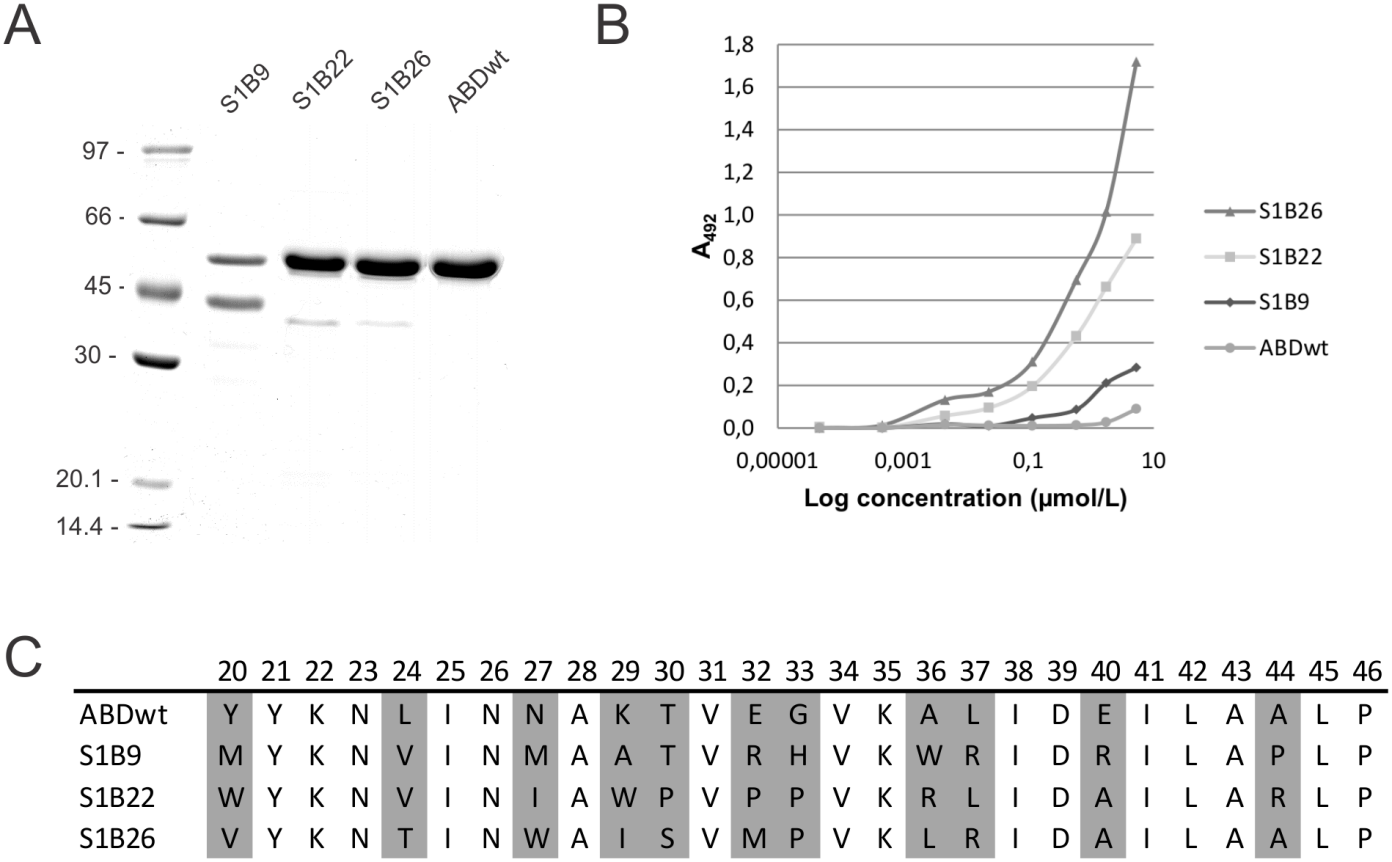
(A) SDS PAGE analysis of selected binders S1B9, S1B22 and S1B26 after purification from *E*. *coli* cell lysates, stained with Coomassie brilliant blue. (B) ELISA-determined binding of serially diluted biotinylated binders selected against immobilized recombinant Stx1B. The binding was detected by HRP-conjugated streptavidin. (C) Sequence similarity comparison of selected binders with parental non-mutated ABDwt. Randomized sequences between residues 20 and 46 were compared. Randomized positions are indicated in grey.

Selected binders in fusion with TolA-Avi and H6 were produced in *E*. *coli* on a large scale, purified with IMAC or HisTrap FPLC and verified on SDS-PAGE electrophoresis, where low level of expression and some proteolytic degradation of S1B9 variant were observed (Fig 3A). Stx1B was immobilized and incubated with serial dilutions of selected S1B binders, detected with HRP-streptavidin. Gradual increase of the ELISA signal confirmed binding of S1B binders to recombinant Stx1B (Fig 3B). Variant S1B26 showed the strongest binding, while binding of S1B9 variant was the weakest, which may be the consequence of degradation noted above. Sequence similarity comparison of selected binders S1B9, S1B22 and S1B26 with parental non-mutated ABDwt is presented in Fig 3C.

Based on ELISA tests and SDS PAGE analysis clones S1B22 and S1B26 were selected for further characterization of binding with surface plasmon resonance (SPR) and microscale thermophoresis (MST), and of stability with thermal shift assay (TSA).

### Characterization of binding affinity of the selected S1B binders

**Fig 4:**
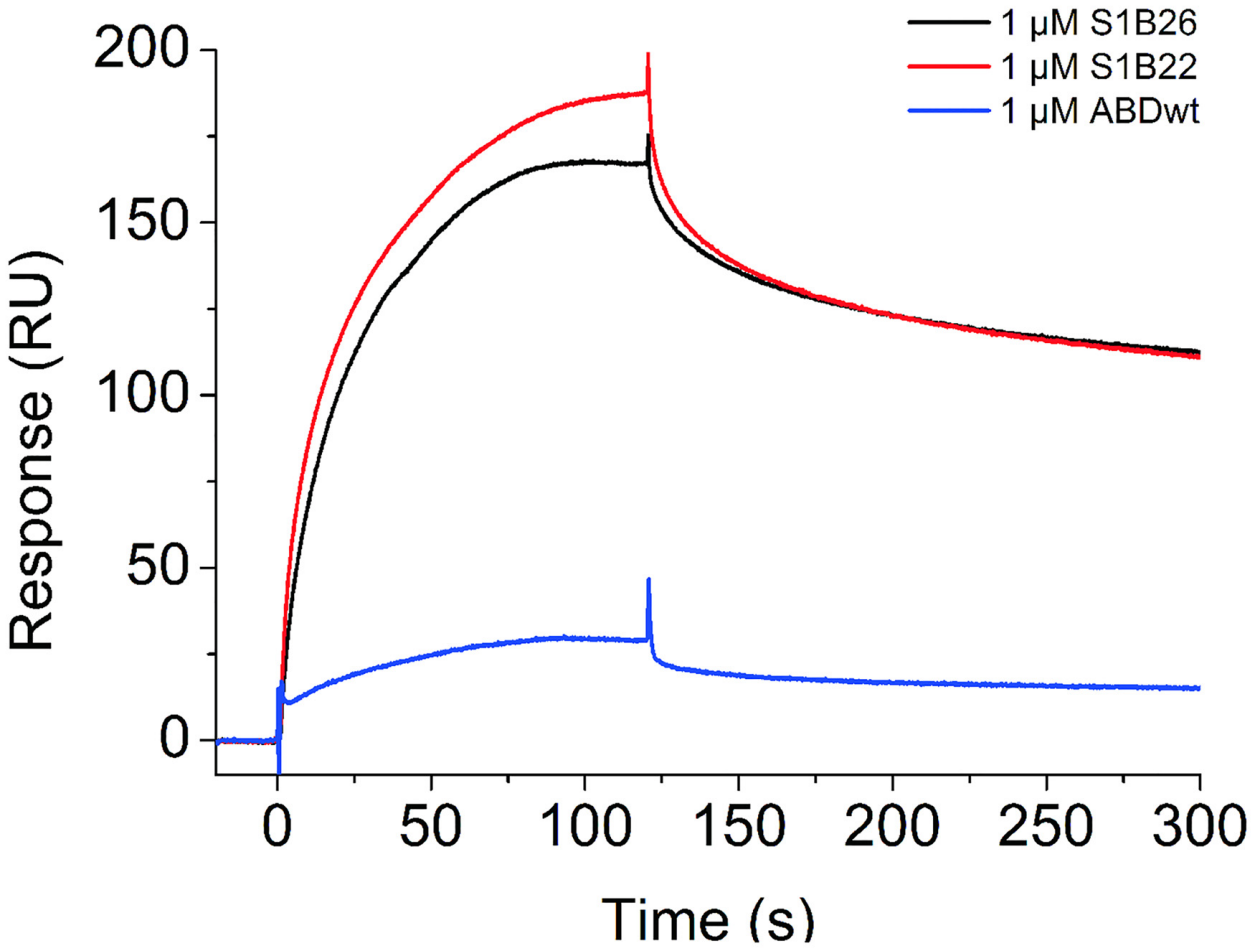
SPR analysis of binding of S1B22, S1B26 and ABDwt in fusion with TolA-Avi and H6 at 1 μM concentration to immobilized recombinant Stx1B.

The first setup of SPR affinity measurements included immobilization of Stx1B on the sensor chip surface and injecting S1B proteins over the surface. Binding was confirmed with different dilutions of both binders, S1B22 and S1B26. Nevertheless, we could not determine the binding constant (*K*_d_) for tested interactions with any of the available binding models in Biacore T100 Evaluation software. This was probably due to a complex interaction between S1B variants and Stx1B, which is in pentameric form, and probably binds more than one S1B molecule. The binding mode between Stx1B and S1B binders could be investigated by structural analysis, such as molecular docking or by solving the structure of the complex. Fig 4 shows a representative response after S1B22, S1B26 and ABDwt, all in fusion with TolA-Avi and H6, were injected at 1 μM concentration over the immobilized Stx1B. A much higher sensor response was observed with S1B22 and S1B26 than with ABDwt.

**Fig 5:**
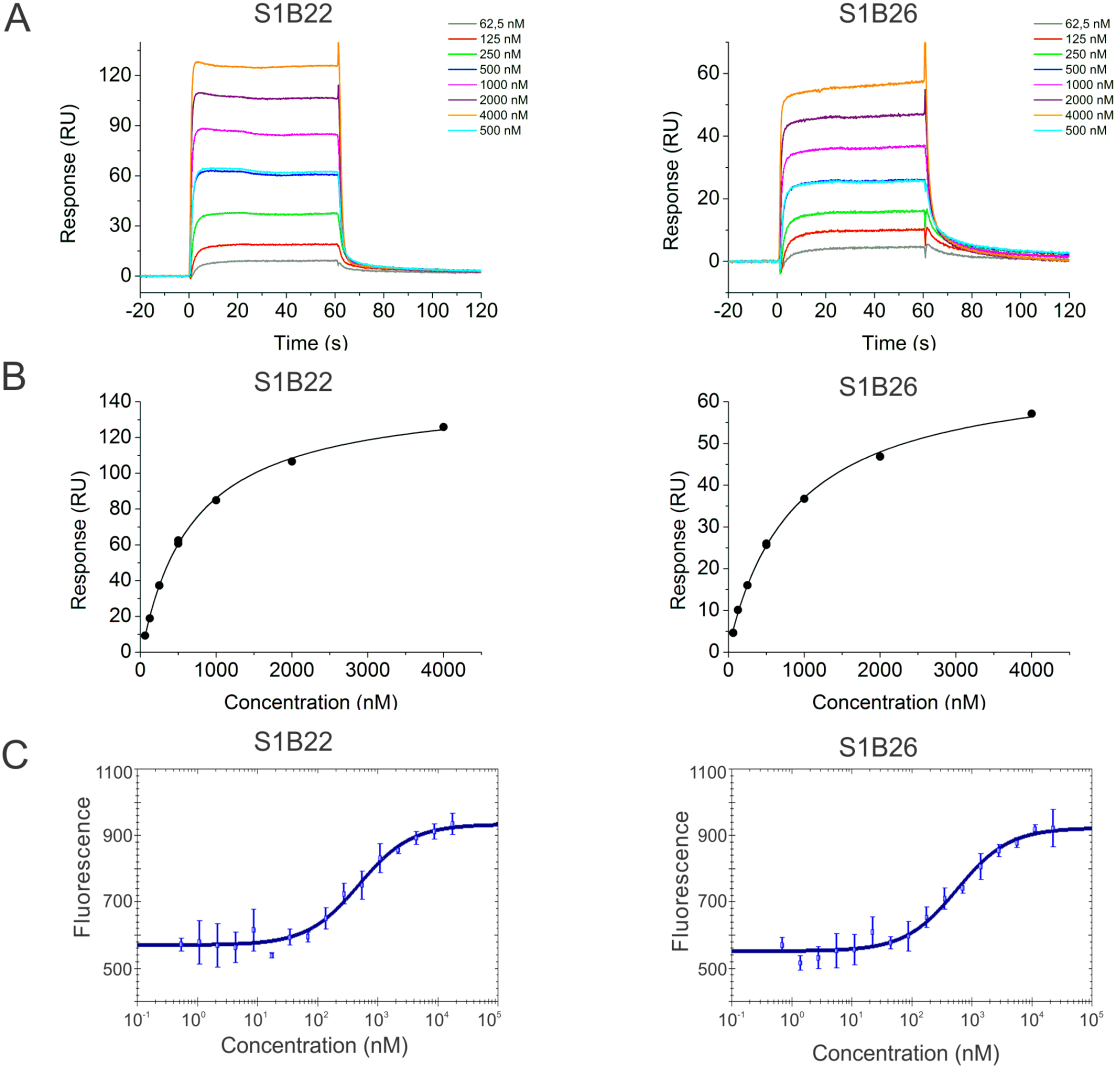
Determination of binding affinity of selected S1B variants to recombinant Stx1B by SPR analysis (A, B) and MST (C). (A) Recombinant Stx1B at seven different concentrations was injected over the chip surface with immobilized H6-S1B22-TolA-Avi or H6-S1B26-TolA-Avi. (B) Steady state response (obtained in (A)) was plotted against Stx1B concentration and Steady State Affinity model was applied to calculate the affinity constant. (C) Sixteen serial dilution concentrations of H6-S1B22-TolA-Avi or H6-S1B26-TolA-Avi were mixed with fluorescently labelled Stx1B at 5 nM final concentration. Fluorescence change *K*_d_ fit in NanoTemper software was used to calculate *K*_d_. Error bars denote standard deviations.

In contrast, by reversing the SPR setup, S1B22 and S1B26 in fusion with TolA-Avi and H6 were attached to the sensor chip surface and two-fold dilutions of recombinant Stx1B, starting with 4 μM concentration, were injected over the chip surface (Fig 5A). Affinity constants were calculated by applying the Steady State Affinity model (Fig 5B) and the average of three experiments was considered. The binding affinity of recombinant Stx1B to S1B22 was 0.70 ± 0.03 μM and, to S1B26, 1.00 ± 0.09 μM.

The binding affinity of S1B22 and S1B26 to fluorescently-labelled recombinant Stx1B in solution was confirmed by MST. The binding curves obtained from three repeat measurements for S1B22 and S1B26 are shown in Fig 5C. The commercial analysis software was used to plot and fit the change in initial fluorescence to yield *K*_d_ values in the micromolar range of 0.4 ± 0.05 μM for S1B22 and 0.6 ± 0.05 μM for S1B26.

Calculated affinities are lower than those reported for ScFvs against Stx1B [38]. However, the affinity of ABDs could be further improved by affinity maturation approach, in which a new combinatorial library would be constructed from the best binder sequence using error prone PCR and where the new library would be subjected to a second selection against the same target under conditions of increasing stringency [40].

### Characterization of the stability of selected S1B binders

The thermal stabilities of binders S1B22 and S1B26 in fusion with TolA-Avi and H6 were investigated by the fluorescence-based thermal shift assay. The denaturation temperature (T_m_) determined for binder S1B26 was 53°C in PBS buffer and 54°C in Tris-NaCl buffer. T_m_ for binder S1B22 could not be determined in any of the tested buffers, probably due to the presence of two tryptophanes and three prolines in the randomized part of S1B22, which are known to destabilize helical structure of proteins. The T_m_ value for parental wild-type ABD has been reported to be 58°C [12, 13].

### Redirection of Stx1B transport in HeLa cells by selected S1B binders

**Fig 6:**
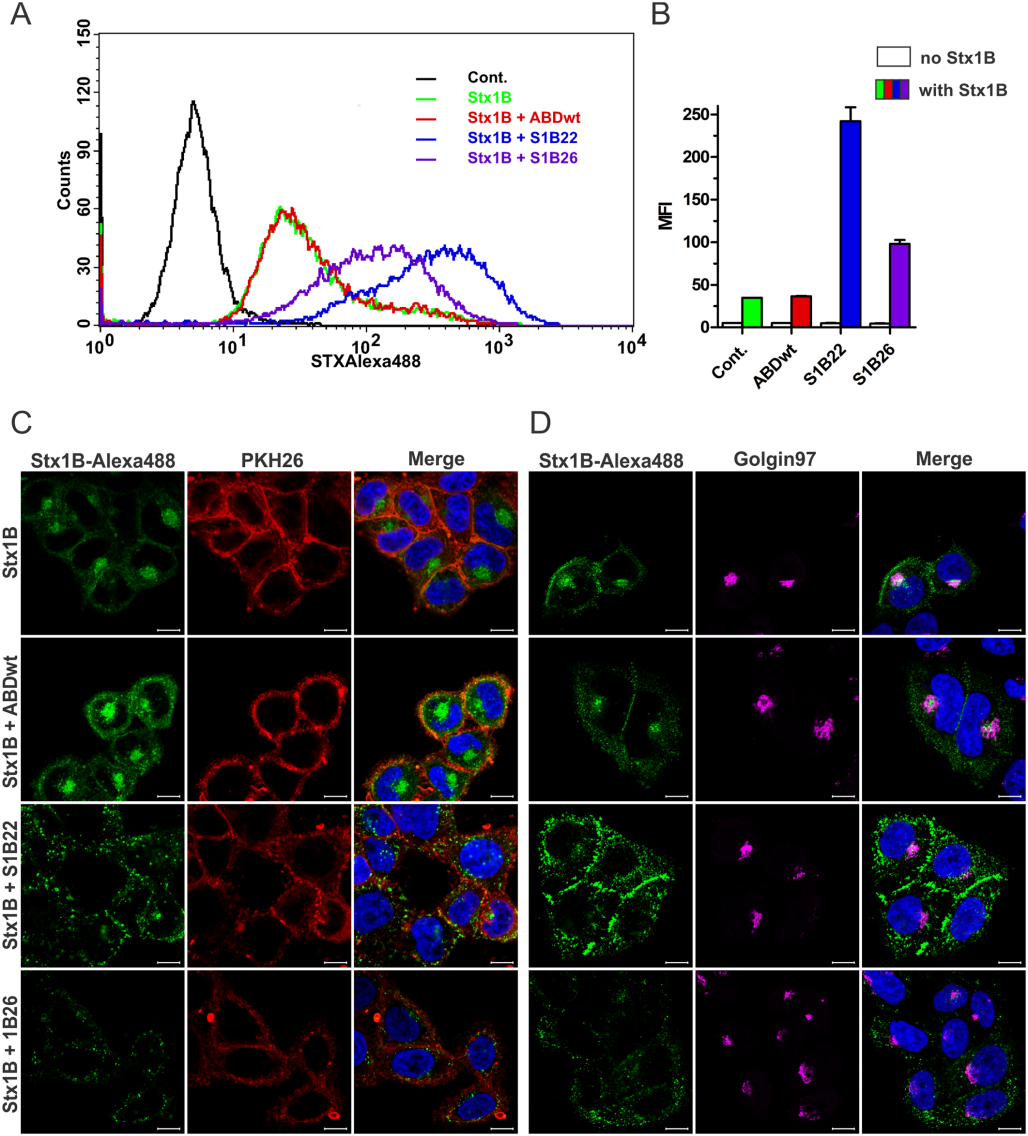
Influence of S1B binders on Stx1B transport into HeLa cells. A, B: Flow cytometric analysis of HeLa cells demonstrating shift in fluorescence intensity (A) and mean fluorescence intensity (MFI; B) upon 1h incubation of HeLa cells with mixtures of S1B22, S1B26 or ABDwt (all in fusion with TolA-Avi and H6) and Alexa Fluor 488-labeled Stx1B. Cont: unstained HeLa cells. Stx1B: HeLa cells incubated with Stx1B-Alexa Fluor 488 alone. C, D: Fluorescence microscopy images of HeLa cells incubated with Alexa Fluor 488 labelled Stx1B (green) with or without pre-incubation with S1B22 and S1B26. DAPI staining (blue) was used to label nuclei. Cells were stained either with PKH26 membrane labeling dye (red; C) and Golgi apparatus was detected with mouse monoclonal Golgin-97 antibody and secondary polyclonal goat anti-mouse antibody conjugated with Alexa Fluor 633 (purple; D). Bars = 10 μm.

S1B22 and S1B26 in fusion with TolA-Avi and H6 were tested for their ability to prevent binding of Stx1B to HeLa cells, or to interfere with retrograde transport of Stx1B to GA in HeLa cells. When fluorescently labelled Stx1B (Stx1B-Alexa Fluor 488) was mixed with S1B binders prior to incubation with HeLa cells, no decrease in mean fluorescence intensity (MFI) compared to the control (incubated with Stx1B-Alexa Fluor 488 alone) was observed with flow cytometry that would be consistent with inhibition of binding to HeLa cells. On the contrary, higher MFI was observed in cells incubated with mixtures of S1Bs and Stx1B-Alexa Fluor 488 (Fig 6A and 6B), indicating higher amount of bound Stx1B-Alexa Fluor 488 or different cell distribution of internalized toxin. ABDwt in fusion with TolA-Avi and H6 had no effect on internalization of Stx1B-Alexa Fluor 488 into HeLa cells (Fig 6A and 6B).

To investigate the effect of S1Bs into more details we performed fluorescence microscopy using membrane labelling marker PKH26 (Fig 6C) and marker for GA (Fig 6D). In cells incubated with Stx1B-Alexa Fluor 488 alone, or mixture of Stx1B-Alexa Fluor 488 and ABDwt, the majority of Stx1B-Alexa Fluor 488 was co-localized with the marker of GA 1 h after addition to HeLa cells (Fig 6D), as shown before with FITC-conjugated Stx1B (Fig 1B). When Stx1B-Alexa Fluor 488 was pre-incubated with S1B22 or S1B26 the distribution of Stx1B in HeLa cells was completely altered. Stx1B-Alexa Fluor 488 was partially co-localized with membrane dye, indicating localization at the cell membrane or in membrane bound vesicles, while the co-localization with the GA marker was completely lacking, indicating its absence from GA. S1B22 and S1B26 interfered with conventional internalization route of Stx1B into the HeLa cells, suggesting a possible inhibitory function of S1B variants on Stx1B retrograde transport. However, more analyses need to be performed to prove the exact mechanism of S1Bs on Stx1B trafficking.

### Expression and surface display of selected S1B binders on *L*. *lactis* NZ9000

**Fig 7:**
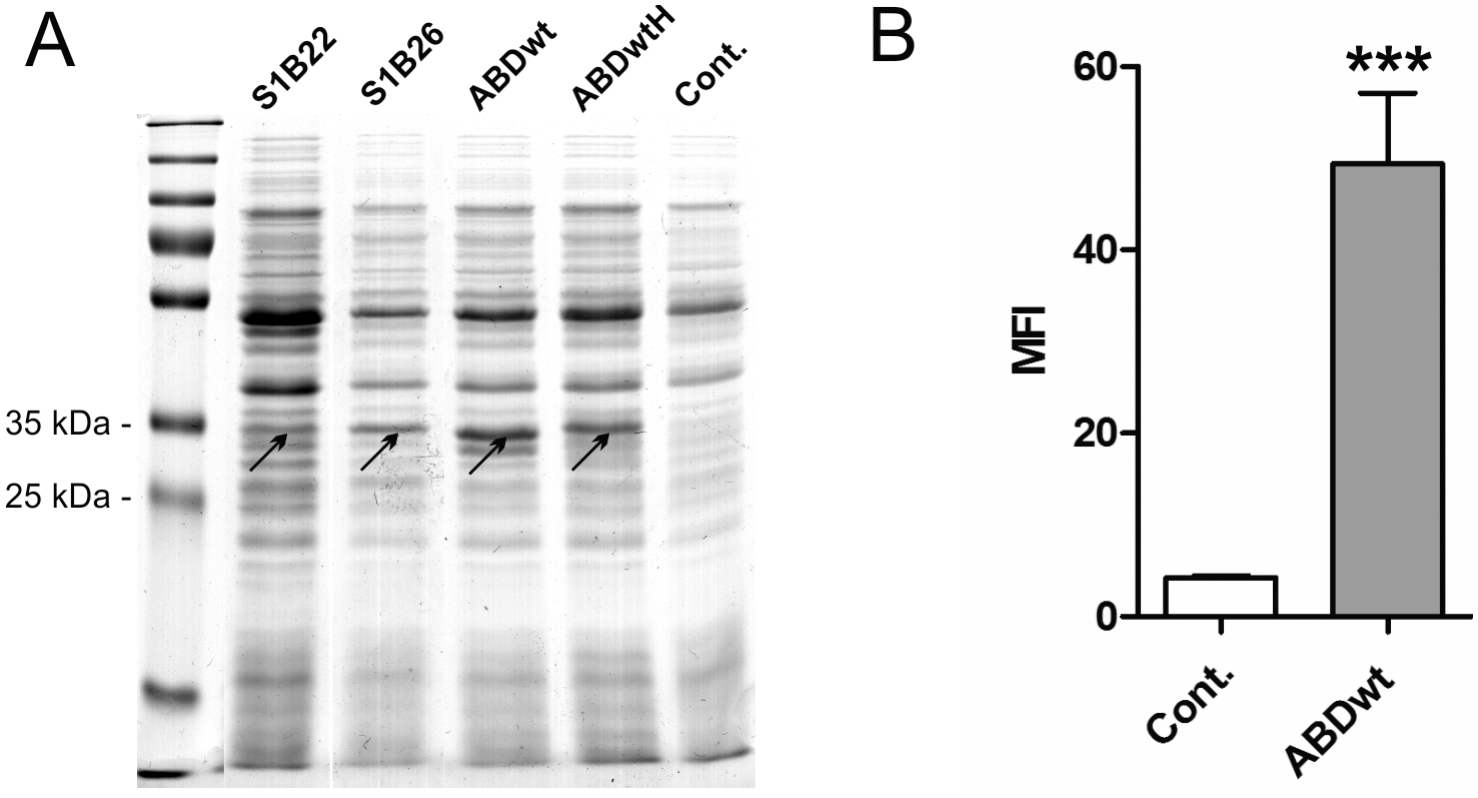
(A) SDS PAGE analysis of lysates of *L*. *lacti*s cells expressing S1B22, S1B26, ABDwt and H6-ABDwt (ABDwtH), all in fusion with Usp45 secretion signal and the LysM-containing cA domain, and stained with Coomassie brilliant blue. ABD fusion proteins are high-lighted with arrows. (B) Flow cytometric analysis of ABD surface display, detection with FITC-conjugated human serum albumin. The MFI value of ABDwt was compared with that of the control using Student’s t test. *** p<0.001. Cont.: control containing empty plasmid pNZ8148.

Genes for S1B22, S1B26, ABDwt and H6-ABDwt without tolA spacer or tags were amplified with PCR and cloned into the plasmid pSDLBA3b [17], which had been designed for the surface display of target proteins in fusion with Usp45 secretion signal [41] and the surface anchoring C-terminal domain of AcmA (cA), as reported [17, 18, 20–22, 33]. Fusion proteins were expressed in *L*. *lactis* by induction with nisin and visualized in the cell lysates by SDS PAGE and Coomassie brilliant blue staining (Fig 7A).

Surface display of ABDs was confirmed for ABDwt and H6-ABDwt using flow cytometry. *L*. *lactis* cells expressing ABDwt on their surface bound FITC-conjugated HSA (Fig 7B), which also indicates the functionality of the displayed binder.

### Evaluation of binding of Stx1B by recombinant *L*. *lactis* with surface displayed S1B binders

**Fig 8:**
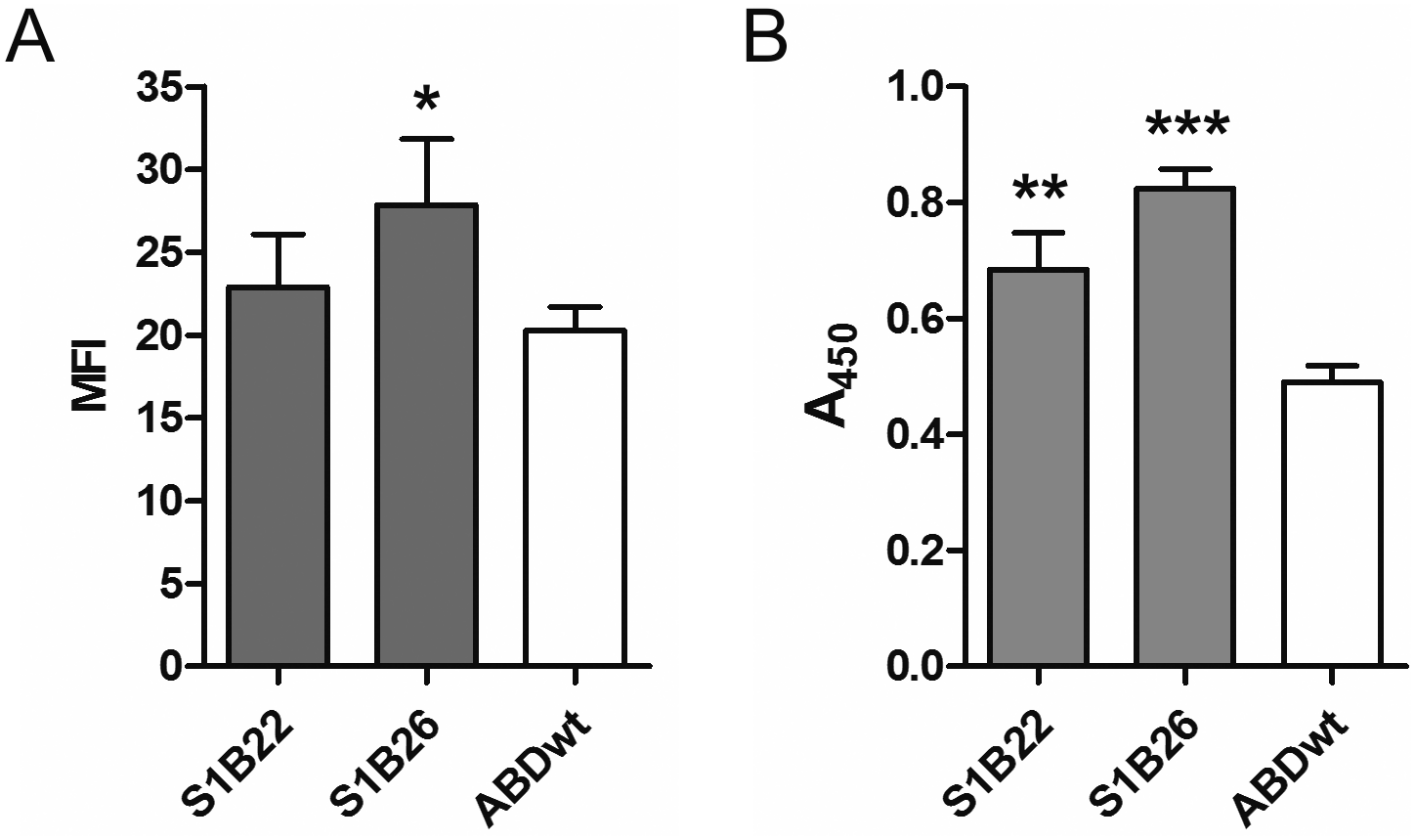
Flow cytometric (A) and whole-cell ELISA (B) analyses of binding of recombinant Stx1B by *L*. *lactis* cells displaying S1B variants or ABDwt on their surface. (A) Alexa488-conjugated Stx1B was used for detection. MFI: Mean fluorescence intensity. (B) Mouse antiStx1B antibody and HRP-conjugated anti mouse antibody were used for detection of Stx1B. A_450_: Absorbance at 450 nm. Vertical bars denote standard deviation. MFI or A_450_ values of S1B binders were compared to those of the ABDwt control using Student’s t test. *: p<0.05, ** p<0.01, *** p<0.001.

Binding of Stx1B by recombinant *L*. *lactis* with surface displayed S1B binders was evaluated by flow cytometry (Fig 8A) and whole cell ELISA (Fig 8B). With flow cytometry, we observed statistically significant binding of fluorescence-labelled Stx1B by *L*. *lactis* cells displaying S1B26 on their surface in comparison with control *L*. *lactis* cells expressing ABDwt. With whole cell ELISA binding of Stx1B by *L*. *lactis* cells displaying both S1B22 and S1B26 was confirmed, the strongest being with S1B26. On the basis of these results, S1B26 was selected as the most promising Stx1B binder for the display on the surface of *L*. *lactis*, even though S1B22 exhibited lower binding constants in SPR and MST, possibly due to the fusion with TolA-Avi and H6 in isolated S1Bs.

## Conclusion

Shiga toxin receptor analogues are an attractive option for treating infections with Shiga toxin-producing bacteria [8]. They place no stress on bacteria and do not induce Shiga toxin release. They bind the toxin *in situ* in the gastrointestinal tract, thereby preventing its activity. Engineered bacteria, such as recombinant *E*. *coli* producing a modified lipopolysaccharide mimicking the Shiga toxin receptor [23, 24], could substitute the polymer analogs. Probiotic food-grade LAB were previously shown to be able to inhibit Shiga toxin-producing bacteria by the production of lactic acid [16] and this could be exploited in creating a synergistic therapeutic effect.

In the present study we demonstrated for the first time functional display of ABD proteins on the surface of LAB *L*. *lactis*. This represents an additional class of alternative binding proteins successfully displayed on the surface of *L*. *lactis*, next to Affibodies [17] and DARPins [18]. LAB *L*. *lactis* capable of binding Stx1B by displaying on its surface ABD-derived Stx1B binding proteins were engineered and could be used for neutralization of Shiga toxin in the human intestine in the early stages of infections with Shiga toxin producing bacteria. Additionally, novel Stx1B binders on the basis of ABD scaffold can be used for basic research as a small non-immunoglobulin alternative to antibodies and with further modification could be developed for diagnostics.
